# RETRACTED: Hwang et al. *Rosa davurica* Pall. Improves *Propionibacterium acnes*-Induced Inflammatory Responses in Mouse Ear Edema Model and Suppresses Pro-Inflammatory Chemokine Production via MAPK and NF-κB Pathways in HaCaT Cells. *Int. J. Mol. Sci.* 2020, *21*, 1717

**DOI:** 10.3390/ijms27073049

**Published:** 2026-03-27

**Authors:** Du Hyeon Hwang, Dong Yeol Lee, Phil-Ok Koh, Hye Ryeon Yang, Changkeun Kang, Euikyung Kim

**Affiliations:** 1 Institute of Animal Medicine, Gyeongsang National University, Jinju 52828, Republic of Korea; pooh9922@hanmail.net (D.H.H.); 2015210922@gnu.ac.kr (H.R.Y.); ckkang@gnu.ac.kr (C.K.); 2 Anti-Aging Research Group, Gyeongnam Oriental Anti-Aging Institute, Sancheong 52230, Republic of Korea; dylee1984@gnoai.or.kr; 3 Department of Anatomy, College of Veterinary Medicine, Gyeongsang National University, Jinju 52828, Republic of Korea; pokoh@gnu.ac.kr; 4 Department of Pharmacology and Toxicology, College of Veterinary Medicine, Gyeongsang National University, Jinju 52828, Republic of Korea

The journal retracts the article “*Rosa davurica* Pall. Improves *Propionibacterium acnes*-Induced Inflammatory Responses in Mouse Ear Edema Model and Suppresses Pro-Inflammatory Chemokine Production via MAPK and NF-κB Pathways in HaCaT Cells” [1] cited above.

Following publication, concerns were brought to the attention of the Editorial Office regarding the integrity of a number of images presented in this publication [1].

In accordance with standard journal procedures, the Editorial Office and Editorial Board conducted an investigation, which resulted in the identification of inappropriate editing and partial duplication within panels presented in Figure 5. Despite numerous attempts to contact the authors and their affiliated institution, no response was received. Consequently, the Editorial Board has lost confidence in the reliability of the findings and has decided to retract this publication, in accordance with MDPI’s retraction policy.

This retraction was approved by the Editor-in-Chief of the *IJMS*.

The authors have been informed of this retraction but did not provide a comment on this decision.

## References

[1] Hwang D.H., Lee D.Y., Koh P.-O., Yang H.R., Kang C., Kim E. (2020). RETRACTED: *Rosa davurica* Pall. Improves *Propionibacterium acnes*-Induced Inflammatory Responses in Mouse Ear Edema Model and Suppresses Pro-Inflammatory Chemokine Production via MAPK and NF-κB Pathways in HaCaT Cells. Int. J. Mol. Sci..

